# The Inhibition of Non-*albicans Candida* Species and Uncommon Yeast Pathogens by Selected Essential Oils and Their Major Compounds

**DOI:** 10.3390/molecules26164937

**Published:** 2021-08-15

**Authors:** Narcisa Mandras, Janira Roana, Daniela Scalas, Simonetta Del Re, Lorenza Cavallo, Valeria Ghisetti, Vivian Tullio

**Affiliations:** 1 Department Public Health and Pediatrics, Microbiology Division, University of Turin, 10126 Turin, Italy; narcisa.mandras@unito.it (N.M.); janira.roana@unito.it (J.R.); lorenza.cavallo@unito.it (L.C.); 2 Department of Veterinary Sciences, University of Turin, 10095 Turin, Italy; daniela.scalas@unito.it; 3 Laboratory of Microbiology and Virology, Amedeo di Savoia Hospital, ASL Città di Torino, 10149 Turin, Italy; simodelre@yahoo.it (S.D.R.); valeria.ghisetti@unito.it (V.G.)

**Keywords:** non-*albicans Candida* species, uncommon yeasts, antifungal activity, essential oils, compounds

## Abstract

The epidemiology of yeast infections and resistance to available antifungal drugs are rapidly increasing, and non-*albicans Candida* species and rare yeast species are increasingly emerging as major opportunistic pathogens. In order to identify new strategies to counter the threat of antimicrobial resistant microorganisms, essential oils (EOs) have become an important potential in the treatment of fungal infections. EOs and their bioactive pure compounds have been found to exhibit a wide range of remarkable biological activities. We investigated the in vitro antifungal activity of nine commercial EOs such as *Thymus vulgaris* (thyme red), *Origanum vulgare* (oregano), *Lavandula vera* (lavender), *Pinus sylvestris* (pine), *Foeniculum vulgare* (fennel), *Melissa officinalis* (lemon balm), *Salvia officinalis* (sage), *Eugenia caryophyllata* (clove) and *Pelargonium asperum* (geranium), and some of their main components (α-pinene, carvacrol, citronellal, eugenol, γ-terpinene, linalool, linalylacetate, terpinen-4-ol, thymol) against non-*albicans Candida* strains and uncommon yeasts. The EOs were analyzed by GC-MS, and their antifungal properties were evaluated by minimum inhibitory concentration and minimum fungicidal concentration parameters, in accordance with CLSI guidelines, with some modifications for EOs. Pine exhibited strong antifungal activity against the selected non-*albicans Candida* isolates and uncommon yeasts. In addition, lemon balm EOs and α-pinene exhibited strong antifungal activity against the selected non-*albicans Candida* yeasts. Thymol inhibited the growth of all uncommon yeasts. These data showed a promising potential application of EOs as natural adjuvant for management of infections by emerging non-*albicans Candida* species and uncommon pathogenic yeasts.

## 1. Introduction

To date, the majority of fungal infections in humans are still due to *Candida albicans* (63–70%). However, other *Candida* species, such as *C. krusei* and *C. parapsilosis*, *Cryptococcus neoformans* and other uncommon yeasts, usually found in the environment and as skin or mucosal colonizers in humans, are emerging as opportunistic pathogens [1,2,3]. Particularly, *C. krusei* is responsible for invasive fungal infections in patients with severely compromised immunity undergoing bone marrow or stem-cell transplantation and in patients with malignant hematological disease [4,5]. *C. parapsilosis* is currently one of the leading species of catheter-related candidemia and fungal infections in neonates and patients in intensive care units. *C. neoformans* generally affects immunocompromised individuals, but patients with no apparent underlying immunodeficiency has been reported in some cases [1,3,6]. Finally, other rare yeast species such as *Sporobolomyces* spp. have been reported as a cause of sporadic invasive bloodstream infections in patients in intensive care units and with central venous catheters, as well as in AIDS patients [6]. Moreover, some common species, such as *Saccharomyces cerevisiae* can be responsible for uncommon clinical cases. This yeast is generally considered a safe organism very useful in agro-alimentary and pharmaceutical fields. However, several isolates have shown virulence features, being able to produce opportunistic infections, mainly in immunodeficient patients [6].

Available antifungal drugs, such as polyenes, triazoles and echinocandins have challenges to cope with the evolving nature of drug-resistant fungal pathogens. Recent trends in acquired antifungal resistance include increased azole resistance among non-*albicans Candida* species isolates, and echinocandin resistance in *C. glabrata*. In addition, some fungal species are intrinsically resistant to certain drugs (e.g., *C. krusei* and fluconazole, *C. neoformans* and echinocandins, or *C. lusitaniae* and amphotericin B). It should be noted that emerging pathogenic yeasts could show resistance to multiple classes of available agents (for example, *C. auris*) [7,8,9]. In order to identify new strategies to counter the threat from antimicrobial-resistant infections (AMRs), including those caused by fungal pathogens, plant-derived compounds have become an important potential for counteracting the therapeutic failures in the treatment of fungal infections.

Amongst phytochemicals, essential oils (EOs) produced by aromatic plants as secondary metabolites and their bioactive pure compounds have been found to exhibit a wide range of remarkable biological activities. Particularly EOs produced by botanical species belonging to Lamiaceae (e.g., *Thymus vulgaris*, *Origanum vulgare*, *Lavandula vera*) and Pinaceae (e.g., *Pinus sylvestris*) are of great pharmaceutical interest. Nevertheless, literature data on the action of the above mentioned EOs against emerging yeast pathogens, except for *C. neoformans*, is still limited, being an open field of new research [3,10]. 

Hence, this work was intended to complete our previous studies on the effectiveness of EOs against less common yeast pathogens [3]. To address this, we investigated the in vitro antifungal activity of nine selected commercial EOs: *T. vulgaris* (thyme red), *O. vulgare* (oregano), *L. vera* (lavender), *P. sylvestris* (pine), *F. vulgare* (fennel), *M. officinalis* (lemon balm), *S. officinalis* (sage), *E. caryophyllata* (clove) and *P. asperum* (geranium), and their main components (α-pinene, carvacrol, citronellal, eugenol, ɣ-terpinene, linalool, linalylacetate, terpinen-4-ol, and thymol) against non-*albicans Candida* strains, *C. neoformans*, and other uncommon yeast pathogens, being a rare cause of invasive infections (*Pichia etchellsii/carsonii*, *Kloechera japonica*, *S. cerevisiae*, *S. salmonicolor*). Fluconazole (FLC) and voriconazole (VRC) were used as reference antifungal agents. 

## 2. Results

In this study, the chemical compositions of *F. vulgare*, *L. vera, M. officinalis*, *P. sylvestris*, *S. officinalis*, *T. vulgaris*, *E. caryophyllata*, *O. vulgare*, and *P. asperum* EOs are presented in Table 1, in according to data gathered from GC-MS and GC-FID phytochemical analyses. 

The MIC and MFC values of EOs, and comparator azole drugs (FLC and VRC) against non-*albicans Candida* species are reported in Table 2. Based on susceptibility testing results, many tested clinical isolates were found to be resistant to FLC (*C. krusei* MIC range = 4–256 mg/mL and *C. norvegensis* MIC range = 32–128 mg/mL), and to VRC (*C. valida* MIC range = 0.06–2 mg/mL and *C. norvegensis* MIC range = 0.25–1 mg/mL).

In our study, pine and lemon balm EOs exhibited strong antifungal activity against the selected non-*albicans Candida* yeast pathogens, with MIC ranges of 0.03–0.12% (*v*/*v*), even against the FLC-resistant and/or VRC-resistant *Candida* strains (*C. krusei*, *C. norvegensis* and *C. valida*, respectively) (Table 2). Other most active EOs were clove, geranium and thyme red (chemotype tymol 26.52%) against *C. krusei*, *C. lusitaniae* and *C. norvegensis* (MIC range 0.06–0.25%, *v*/*v*), whereas oregano, fennel, lavender, and sage were the less effective EOs. 

As can be seen, anticandidal activity of the EOs components against non-*albicans Candida* species was observed only for α-pinene (MIC range 0.002–0.016%, *v*/*v*) while γ-terpinene and linalylacetate did not show any effect on the non-*albicans Candida* species isolates. Carvacrol, eugenol, linalool, and thymol (MIC range 0.06–0.25%, *v*/*v*) were also effective against *C. parapsilosis* and other non-*albicans Candida* species (Table 2).

The results in Table 3 indicate that all EOs and components were considerably potent against all non-*Candida* yeasts. Pine EO exhibited inhibitory effect towards all the tested microorganisms (range 0.004–0.12 %, *v*/*v*). In particularly, this EO displayed the highest inhibitory activity (MIC range 0.015–0.06% *v*/*v*) on *C. neoformans* isolates susceptible to azole drugs. The major component, α-pinene (content 55.7%), was effective against *C. neoformans* and other all non-*Candida* spp., whereas the major component of thyme red, thymol (content 26.5%), effective pure compound, inhibited the growth of all *C. neoformans* isolates at a concentration of 0.002–0.008% (*v*/*v*) than thyme EO alone (MIC range 0.06–0.12% *v*/*v*). Thymol was also more effective on *P. etchellsii/carsonii*, *K. japonica*, *S. cerevisiae* and *S. salmonicolor* (MIC range 0.03–0.12% *v*/*v*). Oregano and thyme red EOs and α-pinene, carvacrol components exhibited slightly lower antimicrobial activity than pine EO (MIC range 0.03–0.12 versus 0.004–0.12% *v*/*v*, respectively) (Table 3).

## 3. Discussion

*Candida* spp. has been reported as the most common cause of fungal infections worldwide [5]. By studying the growing problem of antimicrobial drug resistance, the antimicrobial properties of EOs could be a valuable resource. EOs, as they are known from the literature, are complex mixtures, and the synergistic mechanisms that occur between the components of EOs are important to determine their effects. The biological properties of EOs strictly depend upon their complex phytochemical compositions, which mainly include terpenes (monoterpenes and sesquiterpenes), oxygenated terpenoids (e.g., alcohols and phenols), and other aromatic and aliphatic compounds, at quite different concentrations. As each EO composition varies depending on the plant species and EO chemotype considered, overall bioactivity could be attributed either to the major components of each EO or to combinations of multiple bioactive pure constituents. 

In this study, by GC-MS analysis, the main components constitute for some oils over 50% of the EO and the monoterpenes were the most representative molecules. The terpene profiles of lavender, lemon balm, pine, oregano, clove and thyme EOs in this study were found to be similar to those present in the literature data [4,12,13]. In fact, the phytochemical composition proved to be highly rich in phenolic monoterpenes and only minor differences were observed. 

In our study, pine EO exhibited a high inhibitory activity on the growth of all non-*albicans Candida* isolates with low MIC values ranging from 0.03% to 0.12% (*v*/*v*) and 0.004–0.12% (*v*/*v*) against non-*Candida* yeasts (Table 2). α-pinene, the main component of pine EO, is fairly effective in inhibiting growth stronger than pine EO alone against all tested *Candida* strains, displaying MIC ranging from 0.002% to 0.016% (*v*/*v*) (Table 2) and confirming the good pine EO antimicrobial activity. These findings on α-pinene and pine EO are consistent with previous studies in which many authors reported the good activity of these compounds [3,14]. Moreover, in our previous study on *C. neoformans*, pine EO exhibited a high inhibitory activity on the growth of all azole susceptible isolates, with low MICs [3]. However, our data are in contrast with studies of Motiejūnaite et al. [15] that in 2004 reported that pine EO was very active only against bacteria but not fungi. Although it is known that the compounds making up a larger proportion of the EOs are not necessarily responsible for the majority of the total activity, in this case pine EO activity could be attributed to the presence of major components, such as α-pinene (55.7%). The mechanism by which α-pinene exerts its activity may be due to its action on cell integrity, inhibiting respiration and ion transport processes, and increasing membrane permeability in *C. albicans* [16]. This monoterpene showed different fungistatic or fungicidal activities when tested against non-*albicans Candida* isolates. In particularly, α-pinene exhibited important fungicidal activity against *C. lusitaniae* (Table 2), demonstrating that it would always be advisable to evaluate the activity of oils and components on the single yeast. This finding is in accordance with the report of Salehi et al. [17] who demonstrated that α-pinene has a strong effect against a number of different bacteria and fungi.

The activity of pine EO is particularly high and interesting against non-*Candida* yeasts, i.e., *C. neoformans*, in agreement with Scalas et al. [3], though a number of the ‘rare yeasts’ are encountered as frequent colonizers of human skin and mucosal surfaces [6]. However, in the immunosuppressed host, invasive infections may occur, some being related to non-*Candida* yeasts low pathogenicity. 

In our study all non-*Candida* yeasts were sensitive to the EOs studied (Table 3). In particular, the EO of lemon balm is reported for its high antibacterial and anticandidal activity against the human pathogenic *C. albicans* [18]. Our data showed that this EO exhibited the highest antifungal activity on the non-*albicans Candida* species too. The composition of the oil from *M. officinalis* purchased from Azienda Agricola Aboca includes monoterpene aldehydes and is dominated by citronellal (25.2%) and limonene (12.4%). This composition shows differences with oil composition from different countries in the world [18]. Citronellal displayed antifungal properties against *C. valida* (MIC/MFC = 0.06%, *v*/*v*), probably as a result of make membranes porous [19]. Other studies have shown that the lipophilicity of EOs allows them to separate from the aqueous phase in the membrane structures of fungi, resulting in membrane expansion, and increased membrane fluidity and permeability, alteration of intra-membrane proteins, inhibition of respiration, and alteration of ionic transport processes in fungi and leakage of cellular content [20]. 

Against non-*albicans Candida* isolates, oregano, thyme red (chemotype thymol 26.5%), geranium and clove EOs and carvacrol (62.6% in *O. vulgare* EO), eugenol (82.7% in *E. caryophyllata* EO), linalool (41.9% in *L. vera* EO) and thymol (26.5% in *T. vulgaris* EO) components exhibited a little lower antimicrobial activity than pine and lemon balm EO (range 0.03–0.12%, *v*/*v*) so that the antifungal activity may be attributed to the presence of some elements like carvacrol, eugenol, linalool and thymol which are already well known to exhibit antimicrobial activity (Table 2). The antifungal effects of thyme red EO and thymol were widely reported in literature, and it is one of the ten most commercial oils worldwide [13,21,22,23], but our results showed that is less efficacy than pine. 

Sakkas et al. [13] reported that the antifungal activity of EOs high in carvacrol and thymol depends on their phenolic alcohol content and the antimicrobial potency is determined by their chemical composition. The EO of oregano, rich in carvacrol, inhibited *C. albicans* while the effect of other chemotypes poorer in phenolic components was lower. Our data demonstrated that oregano and carvacrol have interesting antifungal activity against non-*Candida* yeasts. EOs rich in phenols alter the permeability and function of cell membrane proteins. Due to the variety of molecules present in the plant extract, their antimicrobial activity cannot be attributed to a single mechanism but to several mechanisms in the various external and internal cell sites of the microorganism [10,13,24]. 

Additionally, in our study, the EOs have demonstrated potential as antifungal agents when used against non-*albicans Candida* species and non-*Candida* yeasts especially in the presence of yeast resistant to FLC and/or VRC. When the susceptibility profiles among *Candida* spp. were compared, it was observed that *C. norvegensis* isolates were the least susceptible species to FLC and VRC but on the contrary, this yeast was significantly more susceptible to more EOs and components tested.

## 4. Materials and Methods

### 4.1. Essential Oils and Main Components

Commercial EOs of *Foeniculum vulgare* Mill. var. dulce DC, Apiaceae (fennel), *Lavandula* vera DC., Lamiaceae (lavender), *Melissa officinalis* L., Lamiaceae (lemon balm), *Pinus sylvestris* L., Pinaceae (pine), *Salvia officinalis* L., Lamiaceae (sage) and *Thymus vulgaris* L., Lamiaceae, thymol chemotype (thyme red, 26.52% thymol) were purchased from Azienda Agricola Aboca (Sansepolcro, Arezzo, Italy) as steam distilled samples. *Eugenia caryophyllata* Thunb. (*Syzigium aromaticum* L.) Myrtaceae (clove), *Origanum vulgare* L., Lamiaceae (oregano), and *Pelargonium asperum* Willd., Geraniaceae (geranium) were obtained by hydrodistillation and provided by Herboris Orientis Dacor (Milan, Italy).

EO main components (α-pinene, carvacrol, citronellal, eugenol, ɣ-terpinene, linalool, linalylacetate, terpinen-4-ol, and thymol) were purchased from Sigma-Aldrich (Milan, Italy; ≥98% purity) and used as received without any further purification. All samples were protected from light and humidity and stored at 4 °C until use. EO analyses were performed both by gas chromatography with flame-ionization (Agilent Technologies, Waldbronn, Germany) (GC-FID) and by gas chromatography-mass spectrometry (Agilent Technologies) (GC-MS) as previously reported [3,25]. The compounds were identified by the comparison of their linear retention indices (LRIs) relative to C8-C40 n-alkanes (Sigma-Aldrich, Milan, Italy) [26]. Identification of several constituents was carried out by the comparison of their mass spectra with those recorded in the National Institute of Standards and Technology (NIST version 2.0d, 2005) and, when necessary, identification was carried out by co-injection of available reference compounds. The relative percentage amounts of individual components were expressed as the percentage peak area relative to the total composition of the EO obtained by the GC-FID analysis. Quantitative data were acquired as the mean of triplicate analyses for each sample [3]. 

### 4.2. Antifungal Drugs

FLC and VRC powders (≥ 98% purity by HPLC) were purchased from Sigma-Aldrich, Milan, Italy) (n° F8929 and PZ0005, respectively). FLC stock solutions were made up in sterile distilled water, while VRC stock solutions were made up in 100% dimethyl sulfoxide (Sigma-Aldrich, Milan, Italy) and stored at −20 °C until use.

### 4.3. Yeast Isolates

Forty-seven clinical isolates were collected from various specimens (blood, normally sterile body fluids, deep tissue, genital tract, gastrointestinal tract, respiratory tract) from hospitalized patients in Turin (Italy) during 2019. Yeast isolates were sub-cultured onto CHROMagar Candida (Becton Dickinson, Milan, Italy) to ensure purity. Then, they were identified by the API ID32C identification systems (BioMérieux, Rome, Italy), stored at -80 °C in MicrobanksTM (Pro-Lab Diagnostics, Neston, UK), and sub-cultured at least twice on Sabouraud dextrose agar (SDA, Oxoid, Milan, Italy) at 35 °C for 24 h (*Candida* and non-*Candida* spp.). Slowly growing isolates, such as *S. cerevisiae* and *C. neoformans* were incubated for 48–72 h.

### 4.4. Yeast Inocula

Inocula were prepared in sterile saline to reach a 0.5 McFarland turbidity standard and diluted in RPMI 1640 broth medium to a final concentration of 0.5–2.5 × 10^3^ colony forming unit/mL(CFU/mL) [3,25].

### 4.5. Antifungal Susceptibility Testing

Antifungal susceptibility was evaluated by using the Clinical and Laboratory Standards Institute (CLSI M27-A3 and M27-S4) microdilution reference method, with appropriate methodological changes for EOs and EO main components [25,26,27,28,29]. Minimum inhibitory concentration (MIC) determination was performed in RPMI-1640 medium with L-glutamine without sodium bicarbonate (0.2% glucose) (Sigma-Aldrich), buffered to pH 7.0 with 0.165 M morpholinepropanesulfonic acid (MOPS) (Sigma-Aldrich), using 96-well microtiter plates (Sarstedt, Milan, Italy). Stock solutions of EOs and main components were prepared in ethanol (1:2.5) and diluted (1:20) to obtain a final concentration of 2% (*v*/*v*) in RPMI-1640 medium. Tween 80 (Sigma-Aldrich) (final concentration 0.001%, *v*/*v*) was used to enhance EO solubility, with no inhibitory effect on yeast growth.

A 100 μL suspension of final yeast inoculum was transferred into microtiter plates containing 100 μL of two-fold serial dilutions of EOs (range 0.0019–1%, *v*/*v*), or EO components (range 0.0019–1%, *v*/*v*), FLC (range 0.06–128 mg/mL) and VRC (range 0.008–32 mg/mL), respectively. *C. krusei* ATCC 6258 and *C. parapsilosis* ATCC 22019 were included in all the assays as a quality control.

The microtiter plates were incubated at 35 °C for 24 h (*Candida* and non-*Candida* spp.) or 48–72 h (*S. cerevisiae* and *C. neoformans*). EO and EO component MICs were determined visually as the lowest concentration at which no yeast growth was observed. MICs of azoles were determined as the lowest drug concentration that produced ≥50% inhibition of growth relative to growth control. 

CLSI susceptible and resistant breakpoints for FLC were ≤2 and ≥8 mg/mL, respectively, while for VRC were ≤0.12 and ≥1 mg/mL, respectively. Minimum fungicidal concentration (MFC) was determined by subculturing 10 µL of broth taken from all the wells containing either azoles or EO/EO component concentrations without visible growth of yeast onto SDA plates. MFC was defined as a ≥99.9% reduction in the number of CFUs from the starting inoculum count [25,29].

### 4.6. Statistical Analysis

All tests were performed in triplicate in separate experiments. Descriptive statistics were used for MIC, MFC, and modal results were calculated.

## 5. Conclusions

The epidemiology of yeast infections and resistance to available antifungal drugs are rapidly increasing and non-*albicans Candida* species and rare yeast species are increasingly emerging as major opportunistic pathogens. In order to identify new strategies to counter the threat of antimicrobial resistant microorganisms, essential oils (EOs) have become an important potential in the treatment of fungal infections. 

We investigated the in vitro antifungal activity of nine commercial EOs (clove, fennel, geranium, lavender, lemon balm, oregano, pine, thyme red, sage), and some of their main components against non-*albicans Candida* strains and uncommon yeasts. 

Together with our previous data about this topic [3,29], these results show a promising potential application of EOs as natural adjuvants for the management of infections by emerging non-*albicans Candida* species and uncommon pathogenic yeasts, encouraging adequately controlled and randomized clinical investigations.

## Figures and Tables

**Table 1:** Chemical composition of the essential oils.

Components	LRI ^a^	*Foeniculum vulgare* % ^b^	*Lavandula vera* % ^b^	*Melissa* *officinalis* % ^b^	*Pinus sylvestris* % ^b^	*Salvia* *officinalis* % ^b^	*Thymus vulgaris* % ^b^	*Eugenia* *caryophyllata* % ^b^	*Origanum vulgare* % ^b^	*Pelargonium asperum* % ^b^
Anethole	1285	72.1	-	-	-	-	-	-	-	-
Fenchone	1089	14.2	-	-	-	-	-	-	-	-
α-Pinene	933	3.7	-	-	55.7	-	11.5	-	-	-
Eugenol	1358	-	-	-	-	-	-	82.75	-	-
Methyl chavicol	1196	3.7	-	-	-	-	-	-	-	-
Citronellal	1154	-	-	25.2	-	-	-	-	-	25.5
β-Pinene	979	-	-	-	10	-	-	-	-	-
Limonene	1029	3	-	12.4	9.7	-	13.2	-	-	-
Geranial	1270	-	-	8.8	-	-	-	-	-	-
β-Caryophyllene	1425	-	2.9	-	-	-	-	7.9	-	-
Eugenyl acetate	1526	-	-	-	-	-	-	7.1	-	-
p-Cymene	1025	-	-	-	-	-	16.2	-	12.4	-
1,8-Cineole	1032	-	-	-	-	7.7	-	-	-	-
γ-Terpinene	1059	-	-	-	-	-	4	-	7.6	-
Linalool	1099	-	41.9	-	-	-	-	-	-	10.9
α-Terpineol	1192	-	-	6.3	-	-	-	-	-	-
Geraniol	1255	-	-	6.3	-	-	-	-	-	16.2
Neral	1220	-	-	5.4	-	-	-	-	-	-
Camphor	1144	-	-	-	-	22.6	-	-	-	-
trans-Sabinene hydrate	1094	-	-	-	-	29.4	-	-	-	-
Linalyl acetate	1254	-	32.7	-	-	-	-	-	-	-
Lavandulyl acetate	1289	-	3.2	-	-	-	-	-	-	-
Terpinen-4-ol	1179	-	2	-	-	-	-	-	-	-
Carvacrol	1305	-	-	-	-	-	7.8	-	62.6	-
Thymol	1295	-	-	-	-	-	26.52	-	-	-
Citronellyl formate	1352	-	-	-	-	-	-	-	-	5.1
Total % ^b^		96.7	82.7	64.4	75.4	59.7	79.22	97.75	82.6	57.7

^a^ Linear retention index (LRI) calculated on HP-5 column [3,11]. ^b^ Relative percentages of individual components expressed as the % peak area relative to the total composition of the EO obtained by the GC-MS analysis.

**Table 2:** Antifungal activity of EOs (%, *v*/*v*), EO main components (%, *v*/*v*), and comparator antifungal agents (mg/mL) against *Candida* spp.

		*C. krusei* (*n* = 16)	*C. parapsilosis* (*n* = 4)	*C. valida* (*n* = 6)	*C. lusitaniae* (*n* = 2)	*C. norvegensis* (*n* = 4)	All *Candida* spp. (*n* = 32)
Eugenia caryophyllata (Clove)	MIC ^a^	0.06	0.12	0.12	0.06	0.06	
	MIC range	0.06–0.12	0.06–0.25	0.06–0.12	0.06–0.12	0.06–0.12	0.06–0.25
	MFC ^a^	0.12	0.25	0.25	0.12	0.06	
Foeniculum vulgare (Fennel)	MIC ^a^	0.25	0.25	0.5	0.25	0.25	
	MIC range	0.12–0.25	0.25	0.25–1	−/−	0.25–0.5	0.12–1
	MFC ^a^	0.5	0.25	1	0.25	0.25	
Pelargonium asperum (Geranium)	MIC ^a^	0.12	0.12	0.12	0.12	0.25	
	MIC range	0.06–0.12	0.06–0.12	0.12	−/−	0.12–0.25	0.06–0.25
	MFC ^a^	0.12	0.12	0.12	0.12	0.25	
Lavandula vera (Lavender)	MIC ^a^	0.25	0.25	0.5	0.25	0.25	
	MIC range	0.12–0.25	0.25	0.25–0.5	−/−	0.12–0.25	0.12–0.5
	MFC ^a^	0.25	0.5	0.5	0.25	0.25	
Melissa officinalis (Lemon balm)	MIC ^a^	0.06	0.12	0.12	0.06	0.03	
	MIC range	0.03–0.12	0.12	0.03–0.12	−/−	0.03–0.06	0.03–0.12
	MFC ^a^	0.06	0.25	0.25	0.12	0.06	
Origanum vulgare (Oregano)	MIC ^a^	0.12	0.12	0.12	0.12	0.25	
	MIC range	0.12–0.25	0.12–0.25	0.12–0.25	−/−	0.12–0.25	0.12–0.25
	MFC ^a^	0.25	0.25	0.25	0.12	0.25	
Pinus sylvestris (Pine)	MIC ^a^	0.03	0.12	0.06	0.03	0.03	
	MIC range	0.03–0.06	−/−	0.03–0.06	−/−	0.03–0.06	0.03–0.12
	MFC ^a^	0.03	0.12	0.06	0.03	0.06	
Salvia officinalis (Sage)	MIC ^a^	0.5	0.5	0.5	0.5	0.5	
	MIC range	0.25–0.5	0.5–1	0.25–1	−/−	0.25–0.5	0.25–1
	MFC ^a^	0.5	1	1	0.5	0.5	
Thymus vulgaris (Thyme red)	MIC ^a^	0.12	0.5	0.06	0.12	0.06	
	MIC range	0.06–0.12	0.12–0.5	0.06–0.12	−/−	0.06–0.12	0.06–0.5
	MFC ^a^	0.12	0.5	0.12	0.12	0.06	
α-pinene	MIC ^a^	0.002	0.008	0.004	0.002	0.004	
	MIC range	0.002–0.004	0.008–0.016	−/−	−/−	0.004–0.008	0.002–0.016
	MFC ^a^	0.016	0.016	0.008	0.002	0.008	
Carvacrol	MIC ^a^	0.12	0.06	0.12	0.12	0.12	
	MIC range	0.12–0.25	0.06–0.12	0.12–0.25	−/−	−/−	0.06–0.25
	MFC ^a^	0.25	0.12	0.25	0.12	0.12	
Citronellal	MIC ^a^	0.12	0.5	0.06	0.25	0.25	
	MIC range	0.12–0.25	−/−	−/−	−/−	−/−	0.06–0.5
	MFC ^a^	0.25	0.5	0.06	0.25	0.25	
Eugenol	MIC ^a^	0.12	0.06	0.06	0.12	0.12	
	MIC range	0.12–0.25	−/−	0.06–0.12	−/−	0.12–0.25	0.06–0.25
	MFC ^a^	0.25	0.12	0.12	0.12	0.12	
γ-terpinene	MIC ^a^	>1	1	1	>1	>1	
	MIC range	−/−	−/−	−/−	−/−	−/−	1->1
	MFC ^a^	>1	>1	>1	>1	>1	
Linalool	MIC ^a^	0.06	0.06	0.06	0.12	0.12	
	MIC range	0.06–0.25	0.06–0.12	0.06–0.25	−/−	0.12–0.25	0.06–0.25
	MFC ^a^	0.12	0.12	0.12	0.25	0.25	
Linalylacetate	MIC ^a^	1	1	0.5	0.25	1	
	MIC range	−/−	−/−	0.5–1	−/−	−/−	0.25–1
	MFC ^a^	1	1	0.5	0.25	1	
Terpinen-4-ol	MIC ^a^	0.12	0.25	0.12	0.25	0.25	
	MIC range	0.12–0.5	0.25–0.5	0.12–0.25	−/−	0.25–0.5	0.12–0.5
	MFC ^a^	0.25	0.25	0.25	0.25	0.5	
Thymol	MIC ^a^	0.06	0.06	0.12	0.06	0.12	
	MIC range	0.06–0.25	0.06–0.12	0.12–0.25	−/−	0.12–0.25	0.06–0.25
	MFC ^a^	0.12	0.12	0.25	0.25	0.25	
Fluconazole	MIC ^a^	128	0.5	0.12	0.12	128	
	MIC range	4–256 ^b^	0.5–4	0.12–1	−/−	32–128	0.12–256
	MFC ^a^	>256	4	0.12	0.5	>256	
Voriconazole	MIC ^a^	0.06	0.06	0.25	012	0.25	
	MIC range	0.06–1	0.06–1	0.06–2	−/−	0.25–1	0.06–2
	MFC ^a^	0.25	0.12	1	0.12	1	

^a^ MIC and MFC data are presented as modal values (three replicates in three independent assays). ^b^ Despite these values of susceptibility, isolates are assumed to be intrinsically resistant to FLC, and MICs should not be interpreted using breakpoints established by CLSI M27-A3.

**Table 3:** Antifungal activity of EOs (%, *v*/*v*), EO main components (%, *v*/*v*), and comparator antifungal agents (mg/mL) against non-*Candida* yeasts.

		*Cryptococcus neoformans* (*n* = 7)	*Pichia etchellsii/carsonii* (*n* = 2)	*Kloechera japonica* (*n* = 1)	*Saccharomyces cerevisiae* (*n* = 4)	*Sporobolomyces salmonicolor* (*n* = 1)	*Non-Candida* spp. (*n* = 15)
Eugenia caryophyllata (Clove)	MIC ^a^	0.06	0.015	0.12	0.06	0.25	
	MIC range	0.03–0.06	−/−	−/−	0.06–0.12	−/−	0.015–0.25
	MFC ^a^	0.06	0.015	0.12	0.25	0.25	
Foeniculum vulgare (Fennel)	MIC ^a^	0.12	0.12	1	0.25	0.5	
	MIC range	0.12–1	−/−	−/−	0.25–1	−/−	0.12–1
	MFC ^a^	0.25	0.5	>1	1	>1	
Pelargonium asperum (Geranium)	MIC ^a^	0.12	0.25	0.25	0.12	0.25	
	MIC range	0.03–0.12	−/−	−/−	0.12–025	−/−	0.03–0.25
	MFC ^a^	0.12	0.25	0.25	0.25	0.25	
Lavandula vera (Lavender)	MIC ^a^	0.12	0.12	1	0.5	1	
	MIC range	−/−	−/−	−/−	0.12–0.5	−/−	0.12–1
	MFC ^a^	0.12	0.12	1	0.5	1	
Melissa officinalis (Lemon balm)	MIC ^a^	0.12	0.06	0.5	0.06	0.25	
	MIC range	0.06–0.12	−/−	−/−	0.06–0.12	−/−	0.06–0.5
	MFC ^a^	0.12	0.06	0.5	0.12	0.25	
Origanum vulgare (Oregano)	MIC ^a^	0.03	0.03	0.06	0.12	0.06	
	MIC range	0.03–0.06	−/−	−/−	−/−	−/−	0.03–0.12
	MFC ^a^	0.06	0.03	0.06	0.12	0.06	
Pinus sylvestris (Pine)	MIC ^a^	0.015	0.004	0.12	0.03	0.12	
	MIC range	0.015–0.06	−/−	−/−	0.015–0.06	−/−	0.004–0.12
	MFC ^a^	0.015	0.015	0.12	0.06	0.12	
Salvia officinalis (Sage)	MIC ^a^	0.12	0.12	>1	1	>1	
	MIC range	0.12–0.5	−/−	−/−	0.25–1	−/−	0.12–1
	MFC ^a^	0.12	0.25	>1	1	>1	
Thymus vulgaris (Thyme red)	MIC ^a^	0.06	0.06	0.12	0.12	0.12	
	MIC range	0.06–0.12	−/−	−/−	0.03–0.12	−/−	0.03–0.12
	MFC ^a^	0.12	0.06	0.12	0.12	0.12	
α-pinene	MIC ^a^	0.06	0.03	0.03	0.06	0.03	
	MIC range	0.06–0.12	−/−	−/−	0.06–0.12	−/−	0.03–0.12
	MFC ^a^	0.06	0.03	0.03	0.06	0.03	
Carvacrol	MIC ^a^	0.06	0.12	0.03	0.03	0.06	
	MIC range	0.06–0.12	−/−	−/−	−/−	0.06–0.12	0.03–0.12
	MFC ^a^	0.12	0.12	0.03	0.03	0.06	
Citronellal	MIC ^a^	0.5	>1	1	1	>1	
	MIC range	0.25–0.5	−/−	−/−	−/−	−/−	0.25− >1
	MFC ^a^	0.5	>1	>1	>1	>1	
Eugenol	MIC ^a^	0.06	0.06	0.06	0.12	0.12	
	MIC range	0.03–0.12	−/−	−/−	0.12–0.25	0.12–0.25	0.03–0.25
	MFC ^a^	0.06	0.12	0.12	0.25	0.25	
γ-terpinene	MIC ^a^	>1	>1	1	1	>1	
	MIC range	−/−	−/−	−/−	−/−	−/−	1− >1
	MFC ^a^	>1	>1	>1	>1	>1	
Linalool	MIC ^a^	0.5	0.12	0.06	0.12	0.25	
	MIC range	0.5–1	−/−	−/−	0.12–0.25	−/−	0.06–1
	MFC ^a^	0.5	0.12	0.12	0.25	0.25	
Linalylacetate	MIC ^a^	0.12	>1	>1	>1	>1	
	MIC range	0.06–0.12	−/−	−/−	−/−	−/−	0.06– >1
	MFC ^a^	0.12	>1	>1	>1	>1	
Terpinen-4-ol	MIC ^a^	0.06	0.25	0.25	0.5	0.25	
	MIC range	0.03–0.12	−/−	−/−	0.25–0.5	−/−	0.03–0.5
	MFC ^a^	0.12	0.25	0.5	0.5	0.5	
Thymol	MIC ^a^	0.004	0.03	0.06	0.12	0.06	
	MIC range	0.002–0.008	−/−	−/−	0.06–0.12	−/−	0.002–0.12
	MFC ^a^	0.004	0.06	0.06	0.25	0.06	
Fluconazole	MIC ^a^	0.25	0.12	0.25	0.5	0.25	
	MIC range	0.25– >64	0.12–0.25	−/−	0.5–2	−/−	0.12– >64
	MFC ^a^	4	0.5	0.5	2	0.5	
Voriconazole	MIC ^a^	0.015	0.06	0.12	0.25	0.25	
	MIC range	0.015–32	−/−	−/−	0.25–1	−/−	0.015–32
	MFC ^a^	0.06	0.06	0.12	1	0.5	

^a^ MIC and MFC data are presented as modal values (three replicates in three independent assays).

## Data Availability

The data presented in this study are available on request from the first author Narcisa Mandras. The data are not publicly available due to privacy.

## Sample Availability

Samples of EOs fennel, lavender, lemon balm, pine, sage, thyme red, 26.52% thymol, clove, oregano, geranium EOs, (+)-α-pinene, carvacrol, citronellal, eugenol, ɣ-terpinene, linalool, linalylacetate, terpinen-4-ol, and thymol, fluconazole, voriconazole, *Candida* and non-*Candida* spp. strains are available from the authors.
